# Early Steps in Herpes Simplex Virus Infection Blocked by a Proteasome Inhibitor

**DOI:** 10.1128/mBio.00732-19

**Published:** 2019-05-14

**Authors:** Seth M. Schneider, Suzanne M. Pritchard, George A. Wudiri, Chasity E. Trammell, Anthony V. Nicola

**Affiliations:** a Department of Veterinary Microbiology and Pathology, College of Veterinary Medicine, Washington State University, Pullman, Washington, USA; b School of Molecular Biosciences, College of Veterinary Medicine, Washington State University, Pullman, Washington, USA; University of Pittsburgh School of Medicine

**Keywords:** antiviral agents, bortezomib, herpes simplex virus, human herpesviruses, proteasome

## Abstract

Viruses commandeer host cell 26S proteasome activity to promote viral entry, gene expression, replication, assembly, and egress. Proteasomal degradation activity is critical for herpes simplex virus (HSV) infection. The proteasome inhibitor bortezomib (also known as Velcade and PS-341) is a clinically effective antineoplastic drug that is FDA approved for treatment of hematologic malignancies such as multiple myeloma and mantle cell lymphoma. Low nanomolar concentrations of bortezomib inhibited infection by HSV-1, HSV-2, and acyclovir-resistant strains. Inhibition coincided with minimal cytotoxicity. Bortezomib did not affect attachment of HSV to cells or inactivate the virus directly. Bortezomib acted early in HSV infection by perturbing two distinct proteasome-dependent steps that occur within the initial hours of infection: the transport of incoming viral nucleocapsids to the nucleus and the virus-induced disruption of host nuclear domain 10 (ND10) structures. The combination of bortezomib with acyclovir demonstrated synergistic inhibitory effects on HSV infection. Thus, bortezomib is a novel potential therapeutic for HSV with a defined mechanism of action.

## Abstract (precis)

Viruses usurp host cell functions to advance their replicative agenda. HSV relies on cellular proteasome activity for successful infection. Proteasome inhibitors, such as MG132, block HSV infection at multiple stages of the infectious cycle. Targeting host cell processes for antiviral intervention is an unconventional approach that might limit antiviral resistance. Here we demonstrated that the proteasome inhibitor bortezomib, which is a clinically effective cancer drug, has the *in vitro* features of a promising anti-HSV therapeutic. Bortezomib inhibited HSV infection during the first hours of infection at nanomolar concentrations that were minimally cytotoxic. The mechanism of bortezomib’s inhibition of early HSV infection was to halt nucleocapsid transport to the nucleus and to stabilize the ND10 cellular defense complex. Bortezomib and acyclovir acted synergistically to inhibit HSV infection. Overall, we present evidence for the repurposing of bortezomib as a novel antiherpesviral agent and describe specific mechanisms of action.

## INTRODUCTION

Herpes simplex viruses (HSVs) are significant causes of morbidity and mortality in humans worldwide (1, 2). HSV-1 is primarily associated with self-limiting oral mucocutaneous disease and is the leading viral cause of blindness and encephalitis (3–5). Neonatal infections occur in ∼1 in 3,200 deliveries in the United States, and the majority of these infections result in central nervous system disease (6, 7). HSV-2 infection is the most common cause of genital ulcers worldwide (8). Genital herpes increases the risk of acquisition and transmission of human immunodeficiency virus type 1 (HIV-1) (9, 10). HSV causes lifelong latent infection for which there is no cure and no clinically effective vaccine. Acyclovir, the first specific and selective antiviral drug, is a guanosine analogue that targets HSV DNA replication for termination (11). Acyclovir-resistant strains can lead to severe disease, including disseminated infection of immune-dysregulated individuals (12, 13). New, effective therapeutics with different mechanisms of action are needed.

26S proteasomes are ∼2.5-MDa, ATP-dependent multisubunit proteolytic machines present in both the nucleus and the cytoplasm of all eukaryotic cells. They are composed of a barrel-shaped, central 20S core that contains the proteolytic activities. The proteasome executes the controlled degradation of functional proteins as well as the hydrolysis of aberrantly folded polypeptides (14). Proteasome-dependent degradation plays a key role in many cellular processes, such as cell cycle control, proliferation, and apoptosis (15). Viruses can commandeer proteasome activity to promote a diversity of functions critical for their replicative cycles (16). MG132 is a peptide aldehyde that competitively inhibits the degradative activity of the proteasome. Functional proteasomes facilitate HSV entry at a postpenetration stage. MG132 impairs incoming HSV capsid transport to the nuclear periphery (17). Proteasome inhibitors also block HSV infection by preventing degradation of promyelocytic leukemia **(**PML) isoforms, stabilizing nuclear domain 10 (ND10), and, in turn, preventing lytic replication (18–20). Thus, the proteasome is important for early events in HSV infection. Many antiviral drugs are designed to target viral proteins to ensure specificity and avoid toxicity, but such antivirals select for drug-resistant virus mutants. In contrast, antivirals that target cellular proteins required for viral replication, such as proteasomal components, avert the development of resistance.

**FIG 1:**
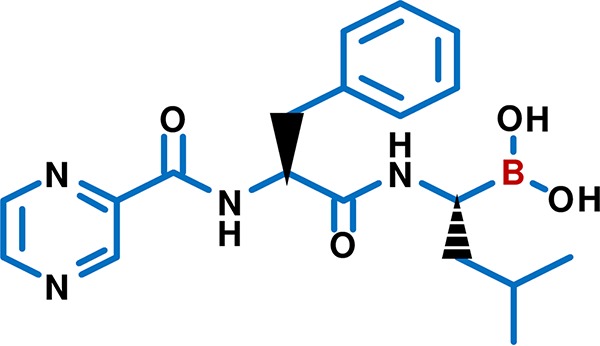
Chemical structure of the proteasome inhibitor bortezomib (C_19_H_25_BN_4_O_4_). Bortezomib [N-(2,3-pyrazine) carbonyl-l-phenylalanine-l-leucine boronic acid] inhibits the proteasome via binding of its boron atom (red) to the chymotrypsin-like active site of the proteasome. The figure was drawn with PubChem Sketcher (103).

Bortezomib [N-(2,3-pyrazine)carbonyl-l-phenylalanine-l-leucine boronic acid] (C_19_H_25_BN_4_O_4_; Fig. 1), originally known as PS-341, is a dipeptide boronic acid inhibitor of the proteasome. Proteasome inhibitors, including bortezomib, trigger apoptosis preferentially in tumor cells and serve as novel anticancer drugs (21). Bortezomib was clinically approved by the U.S. FDA to treat multiple myeloma, an incurable white blood cell cancer, and mantle cell lymphoma, a B-cell non-Hodgkin’s lymphoma, in 2003 and 2006, respectively (22–24). The boron atom (Fig. 1) binds directly to the chymotrypsin-like active site of the proteasome, which is located on the beta-5 subunit of the 20S particle (25).

Here, we report anti-HSV activity of the proteasome inhibitor bortezomib. Overall, we provide evidence that bortezomib inhibits infection of multiple HSV strains early in the infectious cycle, exhibits minimal cytotoxicity, mechanistically halts viral capsid transport to the nucleus and stabilizes ND10 structure, and exhibits synergy with acyclovir.

## RESULTS

### Bortezomib inhibits HSV infection.

**FIG 2:**
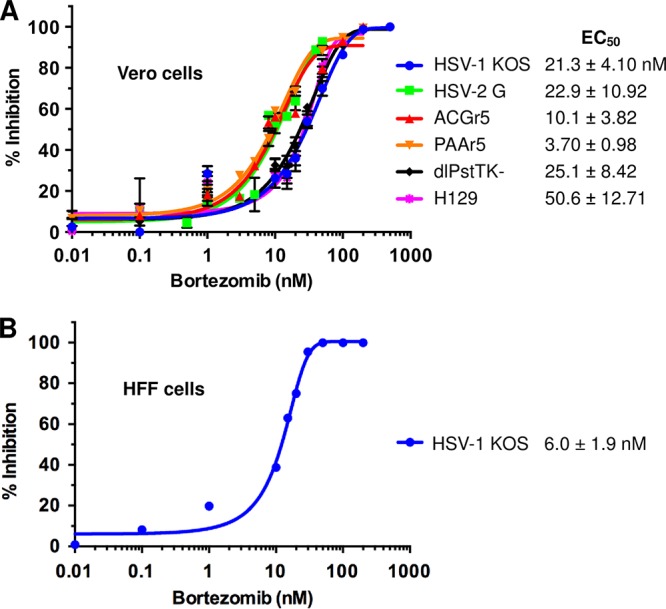
Bortezomib inhibits HSV infection. The indicated strains of HSV were added to (A) Vero cells (MOI of 0.004) or (B) HFF cells (MOI of 0.004) in the presence of increasing concentrations of bortezomib. At 18 to 24 h p.i., cells were fixed and assayed for HSV plaque formation. Plaque reduction is indicated as inhibition represented as a percentage of PFU obtained in the absence of drug. EC_50_ values for each virus as shown were calculated using GraphPad Prism software and range from 3.7 to 50.6 nM. Data are presented as graphed representatives of results from at least three experiments for each strain. Error bars, standard deviations (SD). EC_50_ data are presented as means ± standard errors of the means (SEM).

To determine the effect of bortezomib on HSV infectivity, HSV and increasing concentrations of bortezomib were added to Vero cells. Bortezomib decreased the infectivity of HSV-1 strain KOS and HSV-2 strain G in a dose-dependent manner (Fig. 2A). Bortezomib also reduced the infectivity of acyclovir-resistant HSV-1 strains ACGr5, PAAr5, and *dl*sptk. These laboratory-constructed viruses contain mutations in the HSV thymidine kinase or DNA polymerase genes that render them resistant to acyclovir (26–28). Bortezomib was similarly effective at blocking infection by HSV-1 clinical isolate H129, derived from the brain of a herpes encephalitis patient (29). The concentrations of bortezomib that inhibited 50% of HSV infection (EC_50_) are depicted in Fig. 2 The EC_50_ values ranged from 3.7 to 50.6 nM, indicating that bortezomib, at low nanomolar concentrations, inhibits infection by wild-type, acyclovir-resistant, and clinically isolated HSV. In this assay, the EC_50_ value for acyclovir was 380 nM (see Fig. S1 in the supplemental material), which falls within the broad range of EC_50_s reported for acyclovir. Experiments with bortezomib and primary human foreskin fibroblasts (HFFs) yielded an EC_50_ of 6.0 nM (Fig. 2B).

10.1128/mBio.00732-19.1 FIG S1 Acyclovir inhibition of HSV infection of Vero cells. HSV-1 KOS was added to Vero cells (MOI of 0.004) in the presence of increasing concentrations of acyclovir. Plaques were enumerated as described for Fig. 2. The EC_50_ was 380 nM. Download FIG S1, TIF file, 0.06 MB. Copyright © 2019 Schneider et al. 2019 Schneider et al. This content is distributed under the terms of the Creative Commons Attribution 4.0 International license.

### Bortezomib exhibits low cytotoxicity at concentrations effective against HSV infection.

**FIG 3:**
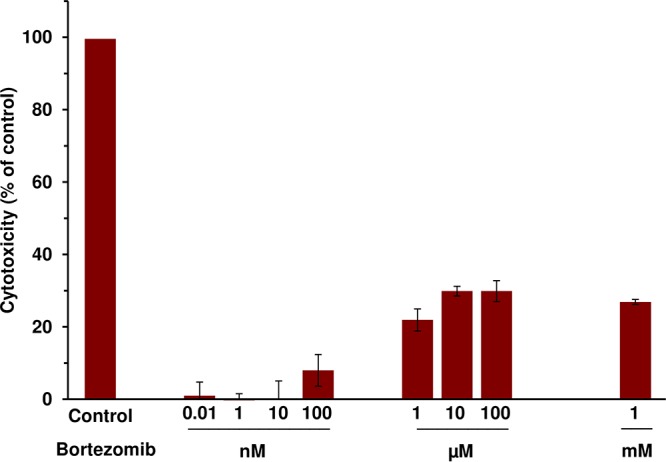
Bortezomib exhibits low cytotoxicity at concentrations effective against HSV infection. Bortezomib was added to Vero cells. At 24 h, LDH activity in the supernatant was assayed as a measure of cytotoxicity. Values are shown as percentages of detergent-lysed control values. Data presented are representative of results from at least three experiments. Error bars, SD.

Proteasome inhibition ultimately leads to cell death (30). Thus, it was important to determine that bortezomib cytotoxicity did not explain the loss in HSV infectivity under our experimental conditions. We exposed Vero cells to doses of bortezomib similar to those utilized in the experiments whose results are presented Fig. 2. The cytotoxicity of bortezomib was quantitated via measurement of the levels of extracellular lactate dehydrogenase (LDH), an enzyme released upon cell death. Bortezomib-induced cytotoxicity peaked at ∼30% at concentrations of >10 µM but did not increase further, even at 1 mM (Fig. 3). Thus, the concentration of bortezomib that is cytotoxic to 50% of cells (CC_50_) is >1 mM. At bortezomib concentrations near the EC_50_ for HSV infection, cytotoxicity levels ranged from 0% to 8% (Fig. 3). Similar results were obtained with human foreskin fibroblasts (Fig. S2). Thus, bortezomib exhibits low cytotoxicity at concentrations that are effective against HSV infection.

10.1128/mBio.00732-19.2 FIG S2 Bortezomib cytotoxicity on HFF cells. Increasing concentrations of bortezomib were added to HFF cells for 24 h, and cytotoxicity was determined via LDH activity. Data presented are representative of results from three experiments. Error bars, SEM. Download FIG S2, TIF file, 0.2 MB. Copyright © 2019 Schneider et al. 2019 Schneider et al. This content is distributed under the terms of the Creative Commons Attribution 4.0 International license.

### Bortezomib is effective when added prior to 3 h postinfection (p.i.).

**FIG 4:**
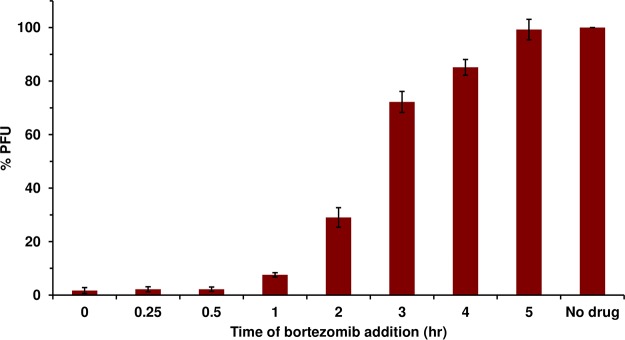
Bortezomib is effective when added prior to 3 h p.i. HSV-1 KOS was added to Vero cells (MOI of 0.001). At the indicated times p.i., 200 nM bortezomib was added. At 18 to 24 h p.i., plaques were enumerated. The values representing mock-treated samples were set to 100%. Data presented are representative of results from three experiments. Error bars, SEM.

To begin to determine the mechanism by which bortezomib blocks HSV infection, a time-of-addition study was implemented. HSV-1 KOS (multiplicity of infection [MOI] of 0.001) was added to Vero cells. At time points from 0 to 6 h p.i., 200 nM bortezomib was added to the infected cells. HSV-1 titers were determined at 24 h p.i. The later bortezomib was added, the less of an effect it had on HSV infection (Fig. 4). Bortezomib added at time zero almost completely inhibited HSV infection but consistently lost effectiveness over time, inhibiting only minimally after 3 h p.i. At least two proteasome-dependent processes occur during the first hours of HSV-1 infection. These results suggest bortezomib acts at an early step of HSV infection that requires the proteasome.

### Bortezomib is not virucidal against HSV.

**FIG 5:**
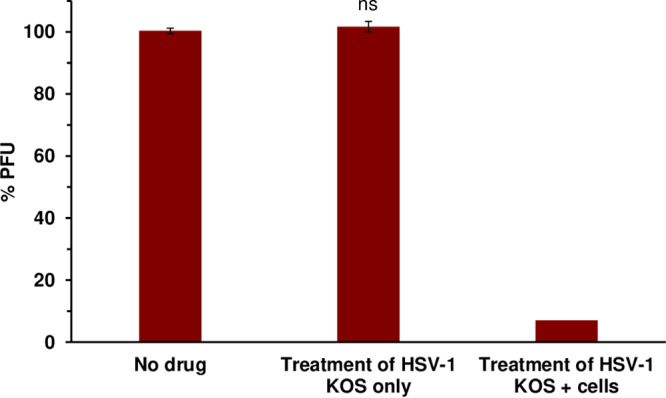
Bortezomib does not exhibit virucidal activity. HSV-1 KOS virions were treated with 100 nM bortezomib at 37°C for 1 h. Bortezomib was diluted to reach noninhibitory concentrations, and titers were determined on Vero cells. Data presented are representative of results from three experiments. Error bars, SEM. ns, not significant (compared to no-drug treatment).

We determined whether the inhibitory effect of bortezomib on HSV infection was due to a direct, inactivating effect on viral particles. HSV-1 KOS was treated with 100 nM bortezomib for 1 h. The virus-drug mixture was diluted to noninhibitory concentrations of bortezomib, and titers were determined on Vero cells. The infectivity of the bortezomib-treated samples was similar to that seen with the vehicle control (Fig. 5). This result suggests that bortezomib does not have a direct, virucidal effect on the infectivity of HSV particles.

### HSV attachment to cells is unaltered by bortezomib.

**FIG 6:**
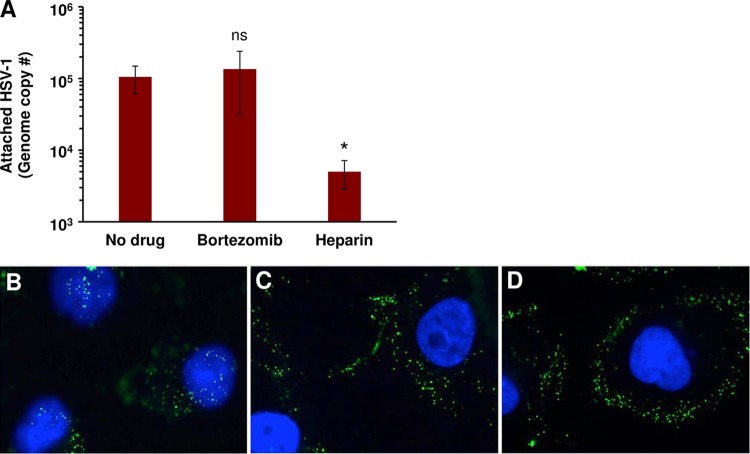
Bortezomib does not affect HSV attachment to cells but inhibits transport of the entering capsid to the nucleus. (A) HSV-1 KOS was added to Vero cells (40 genome copies/cell) in the presence of DMSO control (No drug), 500 nM bortezomib, or 2 µg/ml heparin control. Samples were subjected to spinoculation at 200 × *g* at 4°C for 1 h. After three washes, cell-associated HSV levels were determined by qPCR. Data presented represent means of results from three experiments. Error bars, SEM; ns, not significant; *, *P* value of <0.05 (compared to no drug). (B to D) HSV-1 K26GFP was added to Vero cells on coverslips in the presence of (B) DMSO control or (C) 100 nM bortezomib or (D) 500 nM bortezomib for 2.5 h. Cells were fixed and stained with DAPI nuclear stain and visualized. Data presented are representative of results from at least two experiments.

To rule out the possibility that the bortezomib inhibition was due to an effect on viral attachment to the cell surface, HSV-1 KOS (40 genomes/cell) was added to Vero cells on ice for 1 h at 4°C in the presence of 200 nM bortezomib, a vehicle control, or a heparin treatment control. Cell-attached HSV-1 was quantitated by quantitative PCR (qPCR). Bortezomib-treated HSV-1 attached to cells in a manner similar to that seen with mock-treated HSV-1 (Fig. 6A). Control soluble heparin inhibited HSV-1 attachment to cells by **>**84%. These results suggest that the inhibitory activity of bortezomib is not due to a defect in HSV-1 attachment to cells.

### Transport of the HSV capsid to the nucleus is halted by bortezomib.

Following fusion with a cell membrane, entering HSV nucleocapsids are transported in a proteasome-dependent manner to the nucleus, the site of herpesviral genome replication (17). To determine whether this step in the viral life cycle is affected by bortezomib, HSV-1 K26GFP was added to Vero cells in the presence of 100 or 500 nM bortezomib at 37°C. Herpesviruses utilize multiple entry pathways in a cell-specific manner (31). HSV-1 entry into Vero cells proceeds via direct penetration with the host cell plasma membrane (32, 33). By 2.5 h p.i., in untreated cells, capsids were detected at the nuclear periphery (Fig. 6B). In contrast, in cells treated with bortezomib, HSV-1 capsids were halted at the cell periphery (Fig. 6C and D). Thus, bortezomib impacts HSV-1 infection at an early step, prior to capsid arrival at the nucleus.

### HSV-induced ND10 disruption is prevented in the presence of bortezomib.

**FIG 7:**
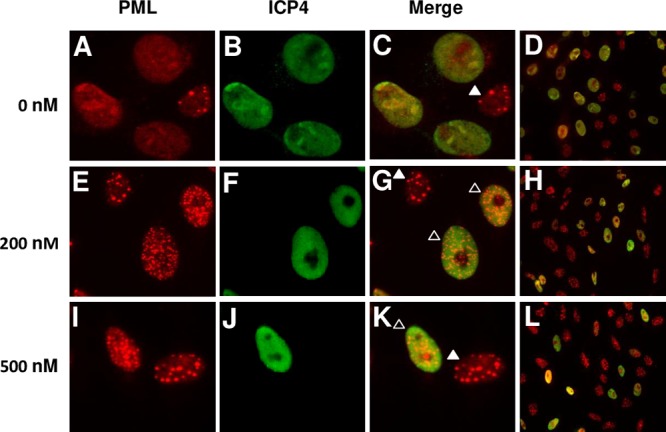
Bortezomib prevents virus-induced ND10 disruption. Vero cells were pretreated with (A to D) DMSO control or (E to H) 200 nM bortezomib or (I to L) 500 nM bortezomib for 15 to 18 min at 37°C. HSV-1 KOS was added to Vero cells (MOI of ∼0.8) for 6 h at 37°C in the continued presence of agent. Cells were fixed, permeabilized, and stained for PML and ICP4. Panels D, H, and L represent zoomed-out views to show more of the surrounding cells. The ICP4 staining results were consistent with ICP4 expressed by the infected cell (37). Data presented are representative of results from three experiments.

Host cell ND10 nuclear bodies contain many proteins responsible for normal functions, including cell cycle regulation, apoptosis, gene transcription, and antiviral defense mechanisms (34–36). A hallmark of infection by viruses, including HSV, is disruption of ND10 (35). HSV-induced disruption of ND10 is proteasome mediated and dependent on viral immediate early protein infected cell protein 0 (ICP0) (20). MG132 halts virus-mediated ND10 dispersal (20). To determine if bortezomib blocks the HSV-triggered dissolution of host ND10, we infected Vero cells with HSV-1 KOS and visualized PML, a major protein component of ND10, via immunofluorescence microscopy. In uninfected, untreated control samples, PML was present in punctate nuclear dots (Fig. S3). Upon infection, confirmed by the presence of HSV-1 ICP4, vehicle control-treated samples lacked punctate dots. Instead, there was diffuse PML throughout the nucleus (Fig. 7A to C), indicating ND10 disruption triggered by HSV infection. The staining in Fig. 7 is consistent with antibody detection of ICP4 expressed by the infected cell (37). Tegument ICP4 is present at ∼150 copies per virion (38) and has not been detected in infected cells. When 200 or 500 nM bortezomib was added prior to infection, the punctate PML staining was sustained during infection (Fig. 7G and K, open triangles). Uninfected (ICP4-negative) cells were also detected when bortezomib was present (Fig. 7G and K, closed triangles), which might have been due to effects of bortezomib on incoming capsid transport. Interestingly, ICP4 staining was still detected in a subset of nuclei, suggesting that viral entry and ICP4 gene expression had occurred while the ND10 structure remained intact (open triangles). Transport of incoming HSV-1 capsids to the nucleus is largely proteasome dependent. However, proteasome-independent transport also occurs and is particularly notable when tegument ICP0 is missing from incoming particles (17, 39). The results represented in Fig. 6 and 7 are consistent with the notion that bortezomib shuts down HSV-1 infection at two steps. If the drug is ineffective at the earlier capsid transport step, it has a second opportunity to inhibit infection by preserving ND10. Overall, these results suggest that bortezomib prevents the disruption of the ND10 host cell defense complex, which is coincident with successful lytic replication of HSV.

10.1128/mBio.00732-19.3 FIG S3 ND10 phenotype in uninfected or HSV-1-infected Vero cells. Mock inoculum (left) or HSV-1 (MOI of ∼0.8; right) was added to Vero cells. At 6 h p.i., cells were fixed, and PML was detected by immunofluorescence to demarcate ND10 structures. ND10 structures appear as nuclear dots in uninfected cells (left). Virus infection-induced ND10 disruption is marked by diffuse nuclear staining of PML in HSV-1-infected cells (right). Download FIG S3, TIF file, 0.5 MB. Copyright © 2019 Schneider et al. 2019 Schneider et al. This content is distributed under the terms of the Creative Commons Attribution 4.0 International license.

### Acyclovir and bortezomib work synergistically to inhibit HSV infection.

**FIG 8:**
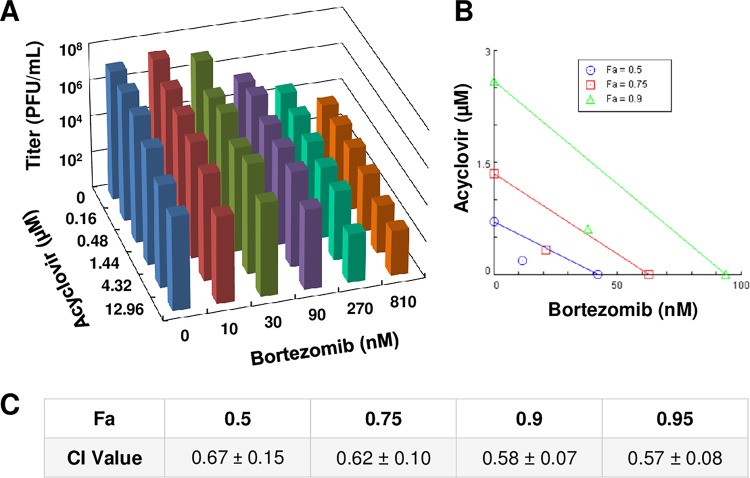
Bortezomib and acyclovir act synergistically to inhibit HSV infection. HSV-1 KOS was added to Vero cells (MOI of 0.1) in the presence of various combinations of acyclovir and bortezomib. At 24 h p.i., cells were fixed, and titers were determined on Vero cells. (A) 3D graph depicting viral titers at the various drug combinations. (B) Isobologram depicting synergistic profiles of bortezomib and acyclovir. “Fa” (fraction affected) refers to fraction inhibition. Each colored line depicts a certain level of fraction inhibition, with endpoints signifying the amount of each drug alone needed to achieve that amount of inhibition. Colored symbols signify how much of each of the two drugs working together is needed to achieve the same inhibition. Symbols below the respective colored lines indicate synergism, those on or near the respective lines indicate additivity, and those above the respective lines indicate antagonism. Data presented are representative of results from three experiments. (C) Software-determined CI values at the specified fractions affected (Fa). Here, “Fa” refers to inhibition of HSV-1 plaque formation (fraction of control). Data are presented as means of results from three experiments ± SEM.

Combination therapies with two or more drugs have the potential to successfully inhibit viral infection more effectively than either drug alone (40). To determine if this is the case for acyclovir and bortezomib, HSV-1 KOS (MOI of 0.1) was added to Vero cells in the presence of acyclovir and bortezomib simultaneously at various concentrations. At 24 h p.i., the cells and supernatant were collected, and the titers of each drug combination sample were determined. Increasing concentrations of either bortezomib alone (topmost bars) or acyclovir alone (blue bars) reduced HSV infectivity (Fig. 8A). Selected combinations of the two drugs also inhibited infectivity. These combinations resulted in minimal cytotoxicity (Fig. S4). Results were analyzed with CompuSyn software, which provided a combination index (CI) value that suggested whether the effect of two drugs was additive, antagonistic, or synergistic. At software-determined concentrations that resulted in 50%, 75%, or 90% inhibition of HSV infection, the CI values were all less than 1, indicating synergy (Fig. 8B and C). CI values for most of the individual concentrations tested were also indicative of synergy, with a minority indicating additivity or antagonism (see Table S1 in the supplemental material). These results suggest that the combination of bortezomib and acyclovir inhibits HSV infection more effectively than treatment with either drug alone, indicative of a synergistic relationship.

10.1128/mBio.00732-19.4 FIG S4 Cytotoxicity profiles of HSV-infected cells treated with combinations of bortezomib and acyclovir. HSV-1 KOS was added to Vero cells (MOI of 0.1) in the presence of various combinations of acyclovir and bortezomib as described for Fig. 8. At 24 h p.i., a sample of supernatant from each condition was assayed for LDH activity as a measure of cytotoxicity. Data represent means of results from three experiments. Download FIG S4, TIF file, 0.3 MB. Copyright © 2019 Schneider et al. 2019 Schneider et al. This content is distributed under the terms of the Creative Commons Attribution 4.0 International license.

10.1128/mBio.00732-19.5 TABLE S1 CI values for a representative acyclovir-bortezomib synergy assay. The superscript 1 in the column 1 and 2 headings indicates that the data represent doses tested on the basis of previously determined EC_50_ values for the two drugs. The superscript 2 in the column 3 heading indicates that the data represent fractions of inhibition of plaque formation. The superscript 3 in the column 4 heading indicates that the data represent combination index (CI) values for the drug combinations as determined via CompuSyn software. CI values of <1 indicate synergy, those of ∼1 indicate additivity, and those of >1 indicate antagonism. Download Table S1, TIF file, 0.3 MB. Copyright © 2019 Schneider et al. 2019 Schneider et al. This content is distributed under the terms of the Creative Commons Attribution 4.0 International license.

## DISCUSSION

The proteasome is a host cell component required for successful infection by HSV. It is needed at multiple steps in the viral life cycle and thus is an attractive target for therapeutic intervention. Targeting a host cell component is expected to decrease the development of viral resistance. Here, we demonstrate that proteasome inhibition by bortezomib effectively inhibits HSV infection in cell culture and identify a mechanism of action. Bortezomib inhibits wild-type, acyclovir-resistant, and clinically isolated HSV infection. It has low cytotoxicity at effective concentrations. Mechanistically, bortezomib acts during the capsid transport and ND10 disruption steps of the HSV life cycle. Bortezomib did not affect HSV attachment to cells and was not virucidal. Bortezomib exhibited synergy with acyclovir. Overall, we present evidence that proteasome inhibition represents an attractive intervention for HSV infection and that the anticancer drug bortezomib might be able to be repurposed as a novel anti-HSV therapeutic.

Incoming HSV-1 nucleocapsid transport to the nucleus is blocked by the proteasome inhibitors lactacystin, epoxomicin, MG132, and bortezomib (17) (Fig. 6C and D). Nuclear delivery of HSV capsids is facilitated by ICP0 present in the virion tegument but is independent of E1 activation of ubiquitin (17, 39, 41). The substrate targeted by the proteasome for HSV capsid transport is not currently known. Nuclear transport of capsids from incoming virions that lack tegument ICP0 is not blocked by proteasome inhibitors; these virions remain infectious in a proteasome-independent manner (39). Our results indicate that while bortezomib blocks the majority of capsid transport to the nucleus, a subset of virions might still enter. HSV-1 ICP4 is detected in nuclei even after bortezomib treatment (Fig. 7). This is consistent with the notion that HSV capsids, while largely dependent on proteasomal activity, can also reach the nucleus via a proteasome-independent mechanism.

Following deposition of the incoming HSV-1 genome into the nucleus, virion subassemblies appear in the vicinity of ND10 structures (42). The mechanism of ND10 antagonism of the viral replicative cycle is not completely understood. However, it likely involves the coordinated action of the major ND10 protein components PML, Sp100, and Daxx, as well as chromatin repression of the viral genome (35, 43). The antiviral activity of host ND10 has been documented for many DNA viruses, including HSV, human cytomegalovirus (HCMV), varicella zoster virus (VZV), Epstein-Barr virus (EBV), Kaposi’s sarcoma-associated herpesvirus (KSHV), adenovirus, human papillomavirus (HPV), and simian virus 40 (SV40) (44). ND10 might also have protective roles during RNA virus infection (44). Dispersal of ND10 structures by herpesvirus infection is thought to facilitate the lytic replication cycle. ND10 disruption typically depends on the proteasome for virus-induced degradation of major ND10 protein components. This is mediated by HSV-1 ICP0 (19, 20), HCMV immediate early 1 (IE1) (45), and EBV BZLF1 (46). Bortezomib blocks ND10 disruption (Fig. 7), as do lactacystin and MG132 (20), supporting the notion that bortezomib acts on another early proteasome-dependent step of HSV infection in addition to capsid transport. Bortezomib blocks infection by vesicular stomatitis virus, influenza virus, hepatitis B virus, Venezuelan equine encephalitis virus, dengue virus, Rift Valley fever virus, Zika virus, African swine fever virus, and Nipah virus (47–56). MG132 inhibits cell infection by the veterinary alphaherpesviruses bovine herpesvirus 1 and pseudorabies virus (57, 58). It is tempting to speculate that bortezomib inhibits other herpesviruses and might, in fact, represent a broad-spectrum antiviral agent.

HSV infection is also facilitated by the proteasome’s degradative activity at later steps in the viral life cycle. Following HSV genome entry into the nucleus, the viral DNA relies partly on nuclear factor kappa-β (NFκ-β) for transcriptional activation (59). Induction of NFκ-β depends on proteasomal degradation of the inhibitor of kappa-β kinase (IκK). Proteasome inhibitors downregulate NFκ-β and, consequently, the HSV transcripts that depend on NFκ-β induction (60). Future efforts will test directly the effect of bortezomib on the expression of HSV-1 immediate early, early, and late genes and the localization of their gene products. HSV ICP0-mediated proteasomal degradation of DNA-dependent protein kinase (61) and centromeric proteins CENP-A (62) and CENP-C (63) has also been described. Proteasome activity is also required during HSV reactivation from latency (64). Here we demonstrate that bortezomib blocks infection by inhibiting two key, proteasome-dependent processes that occur during the first hours of infection: HSV capsid transport to the nucleus (Fig. 6B to D) and ND10 disruption (Fig. 7).

The primary treatment for HSV infection uses the acyclovir family of drugs. Acyclovir is a guanosine analogue that interferes with viral DNA replication. Direct pressure on the viral kinase results in the development of HSV strains resistant to acyclovir (26). Immunocompromised patients are particularly susceptible to infection by acyclovir-resistant strains, which can result in disseminated disease and death (12, 13). Second-line treatments for these severe infections include the use of foscarnet, a pyrophosphate analogue, and of cidofovir, a cytosine analogue, both of which target viral DNA replication (65). Thus, treating acyclovir-resistant strains with these second-line agents might result in the development of multiresistant strains, as has been reported previously for foscarnet (66).

The CC_50_ values for foscarnet and cidofovir on Vero cells are 50 mM and 560 µM, respectively (67, 68). We were unable to reach 50% Vero cell cytotoxicity with concentrations as high as 1 mM. The foscarnet and cidofovir EC_50_ values for HSV-1 on Vero cells are 32.6 µM and 6.4 µM, respectively (69, 70). Bortezomib yields ∼1,000-times-lower EC_50_ values. Moreover, foscarnet and cidofovir both exhibit toxicity *in vivo*, particularly in the form of nephrotoxicity (71, 72). Other side effects of cidofovir include neutropenia and metabolic acidosis (73). The therapeutic dose of foscarnet is 40 to 90 mg/kg of body weight, and its level of toxicity in mice is 500 mg/kg (74). The therapeutic dose of cidofovir is 5 mg/kg, but its level of toxicity in animals is 0.25 to 1 mg/kg (75). The therapeutic dose of bortezomib in cancer treatment is 1.3 mg/m^2^. The level of bortezomib toxicity in animals is 0.6 to 0.9 µg/m^2^ (76). Of course, toxicity in animal models is not always predictive of toxicity in humans. Bortezomib, unlike acyclovir, foscarnet, and cidofovir, targets a host process; thus, antiviral resistance might be less likely.

Host cell processes have been targeted to combat viral infections. Cyclin-dependent kinase (CDK) inhibitors, such as roscovitine, have anti-HSV properties (77, 78), as do cyclooxygenase 2 (COX-2) inhibitors (79). Strategies to inhibit influenza viruses and hepatitis B viruses have also targeted cellular processes (80, 81). Maraviroc is an FDA-approved, clinically effective anti-HIV drug that binds to host chemokine receptors (82–84).

The EC_50_ values for bortezomib against HSV infection ranged from 3.7 to 50.6 nM (Fig. 2). Similar concentrations inhibited proteasome activity and kill cancer cells in culture models of B cell lymphoma and mantle cell lymphoma (85–87). Clinical side effects of bortezomib treatment include peripheral neuropathy (PN) and thrombocytopenia (88, 89). PN might be due to serine protease inhibition by bortezomib in mitochondria, which has a role in the survivability of neurons (90). At a dose of 1.45 to 2.0 mg/m^2^, bortezomib has a half-life of 23 days or 8.7 to 14.8 h in whole blood or plasma, respectively (91, 92). Bortezomib is given twice weekly, and the mean maximum plasma concentration is 200 to 300 nM (92, 93). Thus, the EC_50_s that we obtained were well below the clinically achievable plasma concentration. Therefore, it is possible that plasma concentrations of bortezomib lower than those used for multiple myeloma might be effective against HSV, potentially ameliorating side effects. Bortezomib is clinically effective in patients with immune deficiency resulting from plasma cancers. Thus, bortezomib might be appropriate for administration to the immunocompromised, an important target population for new anti-HSV therapeutics. Bortezomib delivery by subcutaneous administration might be safer than delivery by intravenous administration as suggested by a previous report from a clinical trial in multiple myeloma patients (94). Herpes zoster (shingles), caused by varicella zoster virus, is an adverse event associated with bortezomib use (95). For this reason, acyclovir prophylaxis is recommended for multiple myeloma patients under treatment (96). For the HSV-infected immunocompromised population, acyclovir and low-concentration bortezomib therapy could be imagined and would also reduce the risk of shingles.

Repurposing of existing drugs has been successful in treating other medical conditions. For example, eflornithine is an ornithine decarboxylase inhibitor used to treat African sleeping sickness that was repurposed to treat female facial hirsutism (97). Thalidomide, an anti-inflammatory medication for treatment of leprosy, has recently been approved for treatment of multiple myeloma (98). Bortezomib, like these examples, has already been FDA approved for treatment, which would aid in streamlining the redevelopment process. Altogether, we provide evidence that bortezomib is a novel potential therapeutic for HSV with a defined mechanism of action. The results warrant preclinical testing of bortezomib efficacy in an animal model of HSV infection.

## MATERIALS AND METHODS

### Cells and viruses.

African green monkey kidney (Vero) cells (American Type Culture Collection, Manassas, VA) and human foreskin fibroblasts (HFFs; American Type Culture Collection) were maintained in Dulbecco's modified Eagle's medium (DMEM; Thermo Fisher Scientific, Waltham, MA) containing 10% fetal bovine serum (FBS; Atlanta Biologicals, Atlanta, GA). HSV-1 strain KOS (Priscilla Schaffer, Harvard University); HSV-2 strain G (ATCC); acyclovir-resistant HSV-1 KOS derivatives (Don Coen, Harvard University) ACGr5, containing a mutation in the thymidine kinase gene (99), PAAr5, containing an Arg-to-Ser mutation at residue 842 of the viral DNA polymerase gene (26, 100), and *dl*sptk, containing a 360-bp deletion in the thymidine kinase gene (27); and HSV-1 strain H129 (Richard Dix, Georgia State University), a clinical isolate from the brain of an encephalitis patient (29), were all propagated and their titers determined on Vero cells.

### Chemicals.

Stocks of 50 mM bortezomib (Selleckchem, Houston, TX, or Sigma, St. Louis, MO) and 20 mM acyclovir (Sigma) were prepared in dimethyl sulfoxide (DMSO; Fisher Scientific, Fair Lawn, NJ) and stored at −80°C and −20°C, respectively. Stocks of 0.5 mg/ml heparin (Sigma) were prepared in water and stored at −20°C.

### HSV plaque assay.

HSV-infected Vero cells were incubated at 37°C and 5% CO_2_ for 18 to 24 h. Cells were fixed with methanol-acetone (2:1), dried, and stained with rabbit polyclonal antibody HR50 to HSV (Fitzgerald Industries, Acton, MA) and with horseradish peroxidase-conjugated protein A (Thermo Fisher Scientific). 4-Choloro-1-naphthol substrate (Sigma) and H_2_O_2_ catalyst (VWR International, Inc., Radnor, PA) were added to visualize plaques.

### Cytotoxicity of bortezomib.

Bortezomib or a DMSO vehicle control was added to confluent cell monolayers in 96-well plates. At 24 h, a sample of supernatant was assayed for lactate dehydrogenase (LDH) activity using a Pierce LDH cytotoxicity assay kit (Thermo Scientific, Rockford, IL) according to the manufacturer’s instructions. All concentrations were tested in triplicate. As a positive-control sample, cells were lysed with 1% SDS for 30 min. Cytotoxicity is reported as LDH activity as a percentage of the detergent-lysed sample.

### Time of addition of bortezomib.

HSV-1 strain KOS (MOI of 0.001) was added to Vero cells. Bortezomib or vehicle control was added at a concentration of 200 nM at various times postinfection (p.i.) from 0 to 6 h. Cells were incubated at 37°C and 5% CO_2_ for 18 to 24 h in total and were subjected to HSV plaque assay. Plaques were quantitated, and data are presented as percent inhibition of vehicle control infectivity.

### HSV attachment to cells.

HSV-1 KOS was treated with 2 μg/ml DNase (Turbo DNAFree; Thermo Fisher Scientific) according to the manufacturer's instructions. This treatment removes any free HSV-1 DNA that is not protected inside viral capsids. Virus was diluted in ice-cold binding medium (carbonate-free, serum-free DMEM supplemented with 20 mM HEPES and 0.2% bovine serum albumin [BSA]). Prechilled Vero cells were simultaneously exposed to 200 or 500 nM bortezomib or 2 µg/ml heparin control and virus (40 genome copies/cell). Virus was subjected to spinoculation onto the cells at 200 × *g* for 1 h at 4°C. Cells were washed twice with ice-cold phosphate-buffered saline (PBS; Thermo Fisher Scientific), and cell-associated HSV-1 DNA was isolated with a QIAamp DNA blood minikit (Qiagen, Germany). The HSV-1 ICP22 copy number was determined via qPCR. HSV-1 was quantitated using a CFX96 real-time PCR detection system (Bio-Rad). Primers (Integrated DNA Technologies [IDT], Coralville, IA) were based on KOS ICP22 sequences, both forward (5′ GAG TTT GGG GAG TTT G 3′) and reverse (5′ GGC AGG CGG TGG AGA A 3′) (101, 102). A standard curve was generated for the assay using known quantities of a plasmid containing the HSV-1 ICP22 coding region diluted in glycogen.

### Direct effect of bortezomib on viral particles.

HSV-1 KOS virions (∼3 × 10^7^ PFU) were directly treated with 100 nM bortezomib–culture medium for 1 h at 37°C. Control samples were treated with DMSO vehicle. Samples were diluted 10-fold in culture medium, and HSV-1 titers were determined on Vero cells. The concentration of residual bortezomib in diluted virus preparations (0.01 nM) does not inhibit HSV-1 infection.

### Capsid transport of HSV.

HSV-1 K26GFP (Prashant Desai, Johns Hopkins University) (MOI of ∼30) was added to Vero cells grown on coverslips in the presence of 100 or 500 nM bortezomib and 0.5 mM cycloheximide. At 2.5 h p.i., cultures were washed thrice with PBS and fixed with 3% paraformaldehyde–PBS. Nuclei were counterstained with 5 ng/ml of 4,6–diamidino-2-phenylindole dihydrochloride (DAPI; Roche). Coverslips were mounted on slides with Fluoromount G (Electron Microscopy Sciences, Hatfield, PA) and visualized with a Leica D4000 epifluorescence microscope at ×63 magnification. Images were processed with ImageJ (https://imagej.nih.gov/ij/) and Adobe Photoshop CS5.1.

### Disruption of ND10 nuclear bodies.

Vero cells were seeded on glass coverslips and infected with HSV-1 KOS (MOI of ∼0.8) in the presence of 200 or 500 nM bortezomib or vehicle control. At 6 h p.i., cultures were fixed with 3% paraformaldehyde, quenched with 50 mM ammonium chloride, and permeabilized with 0.1% Triton X-100. Coverslips were stained for ND10 with 1:500 rabbit polyclonal primary antibody against PML (Santa Cruz Biotechnology, Dallas, TX) and 1:1,000 goat-anti-rabbit Cy3 secondary antibody (Thermo Fisher Scientific) and were stained for HSV-1 infection with 1:1,000 mouse monoclonal primary antibody against ICP4 (H1A021; Santa Cruz Biotechnology) and 1:1,000 goat-anti-mouse Alexa Fluor 488 secondary antibody. Nuclei were counterstained with DAPI. Coverslips were mounted onto slides with Fluoromount G and visualized with a Leica D4000 microscope (magnification, ×40). Images were processed with ImageJ (https://imagej.nih.gov/ij/) and Adobe Photoshop CS5.1.

### Synergy of bortezomib and acyclovir.

Vero cells were infected with HSV-1 KOS (MOI of 0.1) and exposed simultaneously to various combinations of acyclovir and bortezomib. Cells were incubated at 37°C and 5% CO_2_ for 24 h. A sample of supernatant was taken for LDH cytotoxicity analysis as described above. Cells were collected and the titers determined via HSV plaque assay as described above. Data are presented as percent inhibition compared to an untreated control. The three-dimensional (3D) graph was constructed using Microsoft Excel. Combination indices and isobolograms were generated with CompuSyn software.

### Ethics statement.

Low-passage-number HSV-1 isolate H129 was part of an already-existing, publicly available collection (26). Institutional review board (IRB) approval was not sought. Samples were anonymized in the previous study.
