# Supplementary figures and images for: Early Steps in Herpes Simplex Virus Infection Blocked by a Proteasome Inhibitor
Source: mBio. 2019 May 14;10(3):e00732-19. doi: 10.1128/mBio.00732-19 (PMC6520451; doi:10.1128/mBio.00732-19)

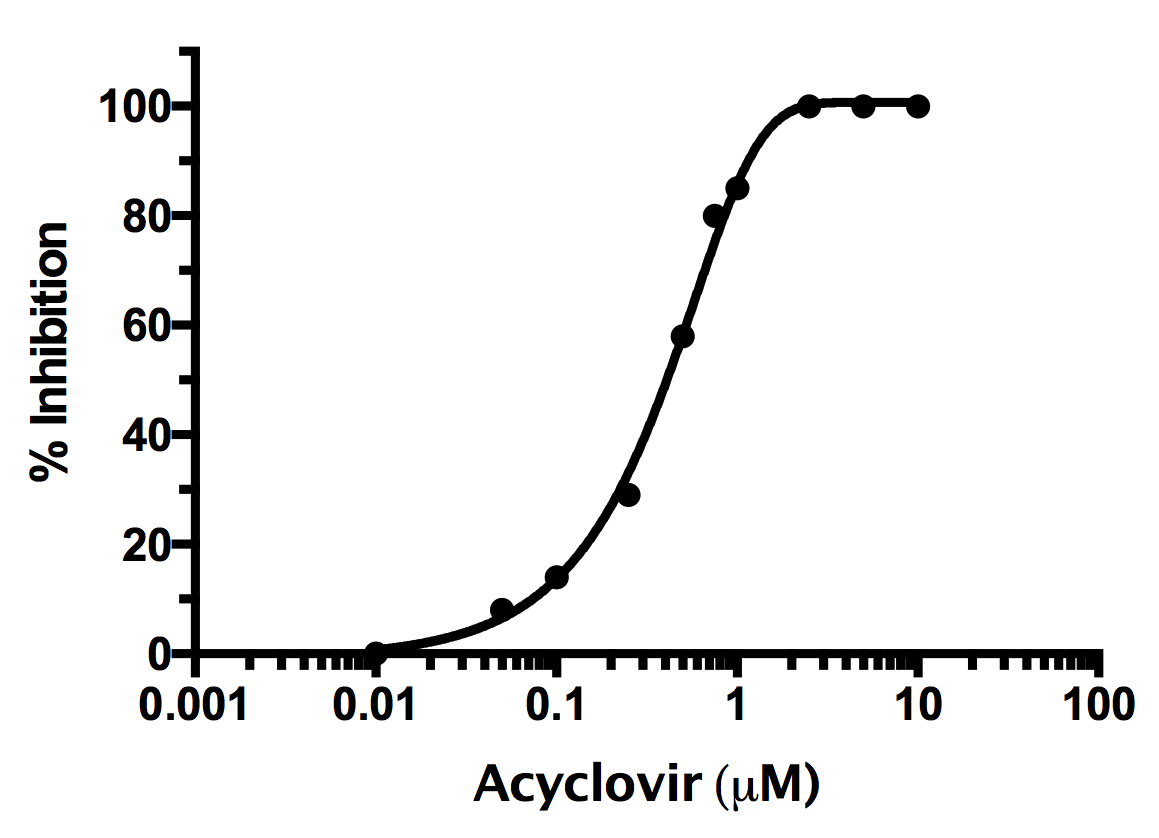

Supplement: FIG S1 [file mBio.00732-19-sf001.tif]

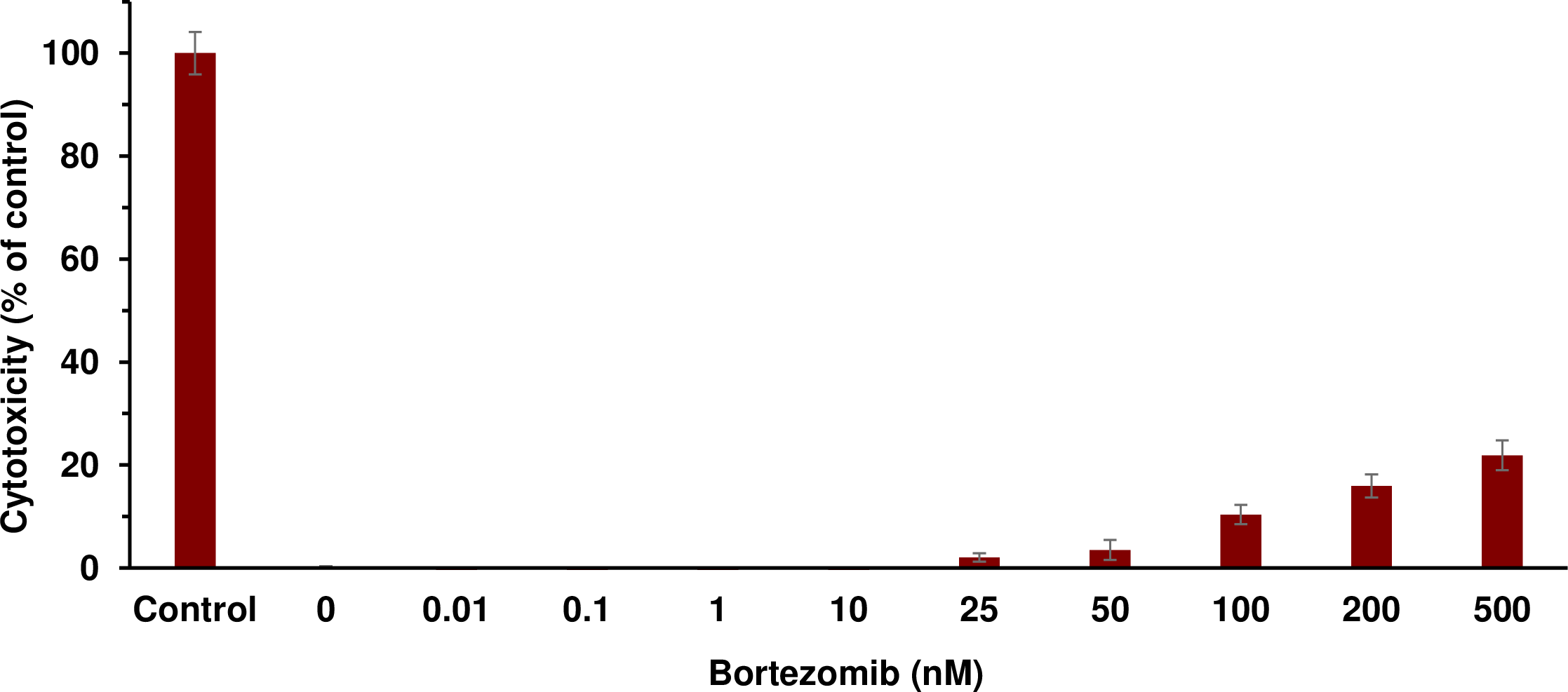

Supplement: FIG S2 [file mBio.00732-19-sf002.tif]

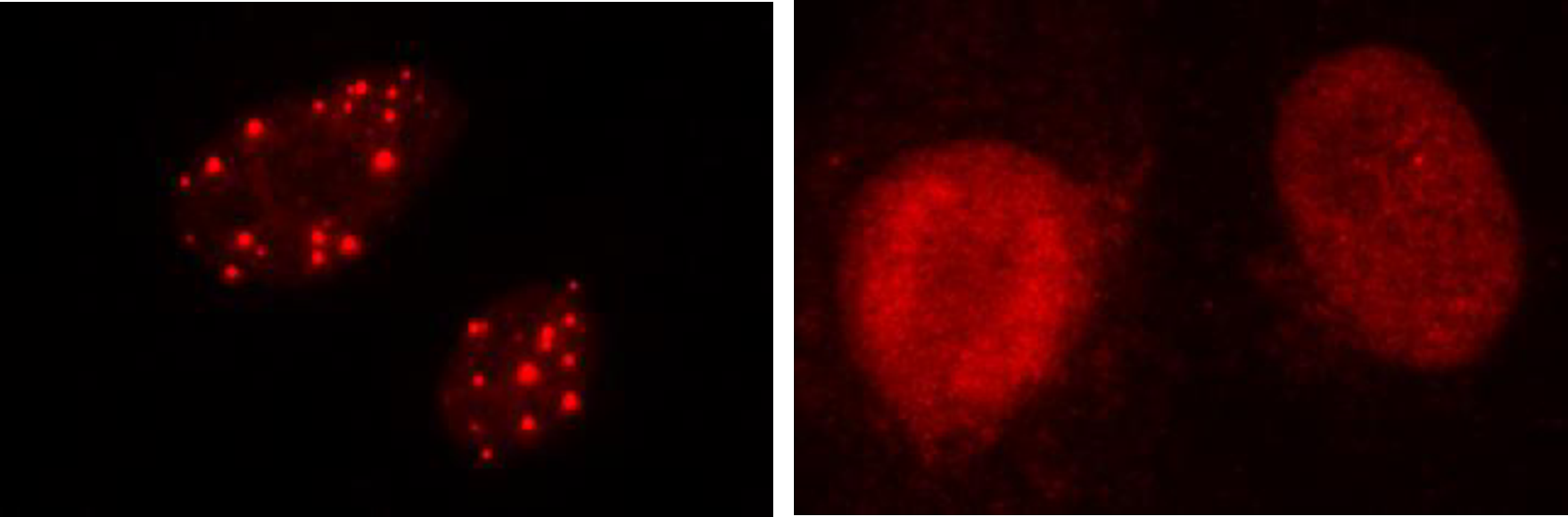

Supplement: FIG S3 [file mBio.00732-19-sf003.tif]

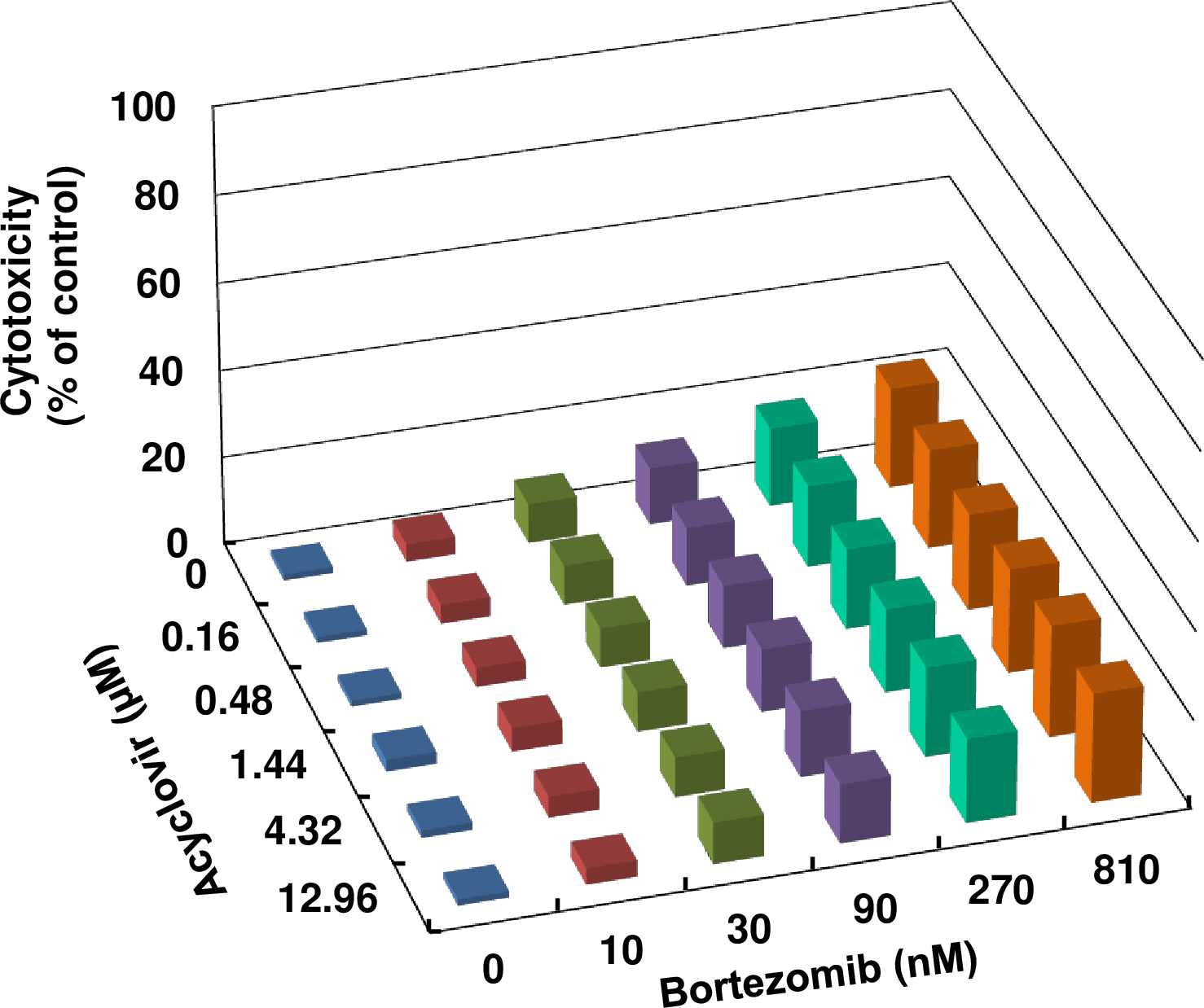

Supplement: FIG S4 [file mBio.00732-19-sf004.tif]

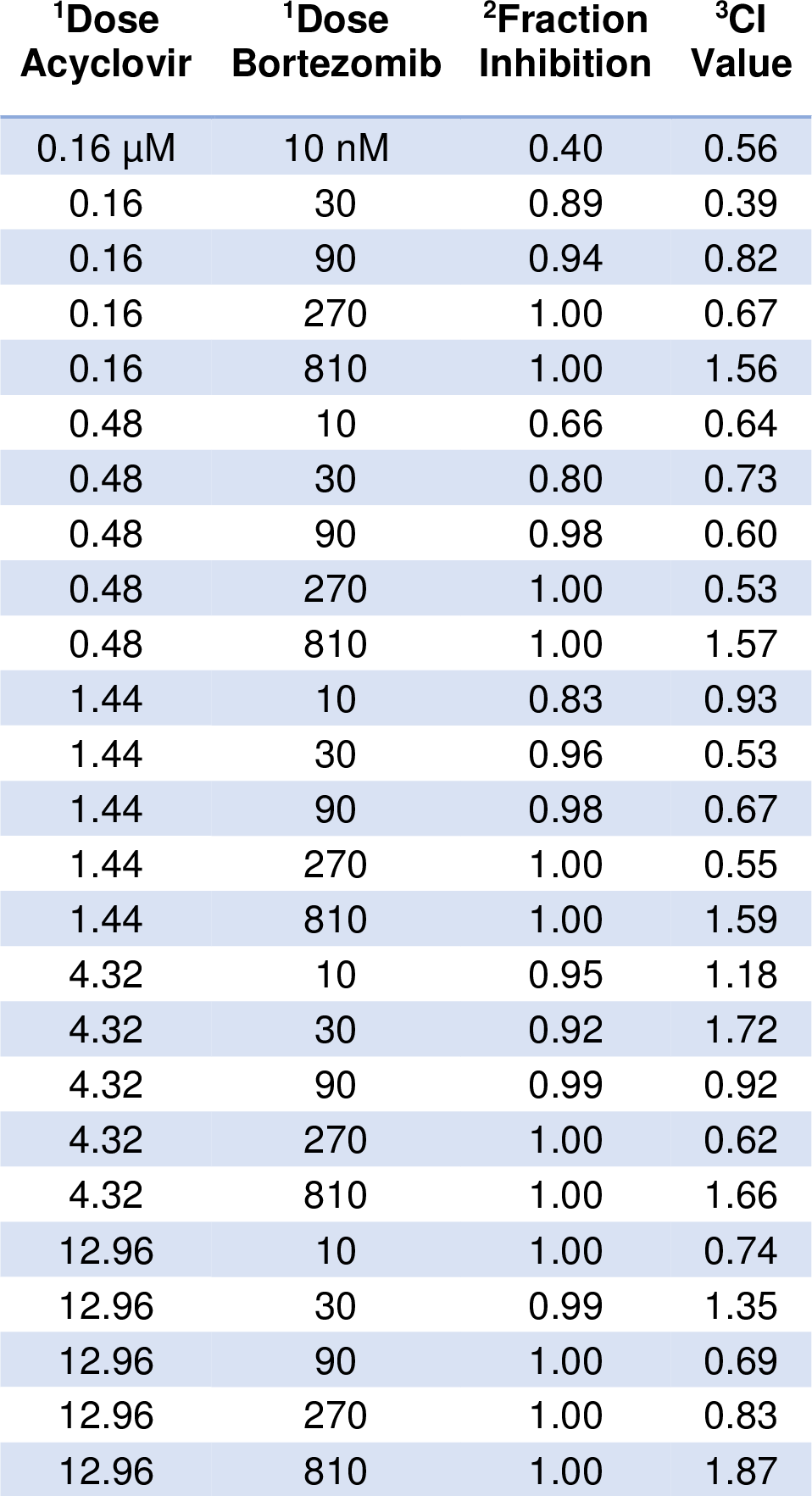

Supplement: TABLE S1 [file mBio.00732-19-st001.tif]
